# USP43 directly regulates ZEB1 protein, mediating proliferation and metastasis of colorectal cancer

**DOI:** 10.7150/jca.48056

**Published:** 2021-01-01

**Authors:** Dao-xiong Ye, Si-si Wang, Ying Huang, Xiao-jie Wang, Pan Chi

**Affiliations:** Department of General Surgery, Fujian Medical University Union Hospital, Fuzhou, Fujian Province, 350001, China.

**Keywords:** colorectal cancer, EMT, ZEB1, USP43, qRT-PCR

## Abstract

Colorectal cancer is one of the most common malignant tumors of the digestive tract. In this study, we had examined the biological role of USP43 in colorectal cancer. USP43 protein and mRNA abundance in clinical tissues and five cell lines were analyzed with quantitative real-time PCR test (qRT-PCR) and western blot. USP43 overexpression treated DLD1 cells and USP43 knockdown treated SW480 cells were used to study cell proliferation, migration, colony formation, invasion, and the expression of epithelial-mesenchymal transformation (EMT) biomarkers. Moreover, ubiquitination related ZEB1 degradation was studied with qRT-PCR and western blot. The relationships between USP43 and ZEB1 were investigated with western blot, co-immunoprecipitation, migration, and invasion. USP43 was highly expressed in colorectal cancer tissues. USP43 overexpression and knockdown treatments could affect cell proliferation, colony formation, migration, invasion, and the expression of EMT associated biomarkers. Moreover, USP43 can regulate ZEB1 degradation through ubiquitination pathway. USP43 could promote the proliferation, migration, and invasion of colorectal cancer. Meanwhile, USP43 can deubiquitinate and stabilize the ZEB1 protein, which plays an important role in the function of colorectal cancer.

## Introduction

Colorectal cancer is one of the most common malignant tumors of the digestive tract and currently ranks third in the incidence of malignant tumors worldwide. Depending on the stage of the tumor, the 5-year survival rate of colorectal cancer is between 10-90% 1. The rectum is the most susceptible site for tumors in the entire large intestine. In western countries, rectal cancer accounts for approximately 40% of the total incidence of colorectal cancer 2. In China, the total incidence of rectal cancer is 60% -70% 3. Among them, the middle and low rectal cancers below the peritoneal reflex plane account for the majority. Rectal cancer has a variety of its pathological characteristics, such as a deep pelvic cavity, the operation is more difficult. Compared with colon cancer, rectal cancer has a high local recurrence rate and low long-term survival rate 4. Moreover, the prognosis of the local progression of rectal cancer is worse, and its 5-year survival rate is between 20-40%. Studies have shown that local recurrence and overall survival rate of colorectal cancer after surgery are related to a variety of factors, such as the type of tumor itself, the degree of cell differentiation, the degree of tumor invasion, location, lymph node metastasis, surgical operation, and comprehensive treatment of the tumor 5. Therefore, how to effectively prevent local recurrence after colorectal cancer surgery and improve the overall survival rate and disease-free survival rate of colorectal cancer patients has been the focus of colorectal cancer research.

The human genome encodes more than 100 types of deubiquitinase enzymes (DUBs), which can be divided into six families 6: 1) the ubiquitin-specific protease family (Ubiquitin-specific proteases, USPs), 2) the ubiquitin post-terminal hydrolase family (Ubiquitin-COOH-terminal hydrolases, UCHs), 3) ovarian tumor-associated protease family (Ovarian tumor proteases, OTUs), 4) Josephins (the family currently only finds a member of Ataxin-3), 5) JAB1/MPN/MOV34 protease family (JAMMS), and 6) Motif interacting with Ub-containing novel DUB family) 7. Among them, the USPs family belongs to cysteine protease, which is the most widely known member of DUBs and the most diverse structure and function. There have been reports in the literature on the relationship between various USPs and tumor development, including USP7, USP21, USP22, USP33, USP39, USP54, etc. 8. For example, USP33 has been shown to interact with the von Hipppel-Lindau (VHL) tumor suppressor protein. Therefore, USP33 is also known as VHL-related deubiquitinating enzyme 1 9. Currently, known substrates of USP33 include β-arestin, Robol, and CPH0 10. USP33 can participate in the regulation of the biological processes of β2AR endocytosis and centrosome synthesis 11. Furthermore, USP33 has also been shown to play a key role in tumorigenesis and development 12. The above evidence showed the important roles of USPs in tumor formation and development. Meanwhile, previous study indicated that USP43 could remarkably inhibit the growth and metastasis of breast cancer though affecting EGFR/PI3K/AKT 13. In addition, USP43 could promote tumorigenesis by regulating cell cycle and EMT process in breast cancer 14. However, there is still poor evidence about the role of USP43 in colorectal cancer.

ZEB1 is an important member of the ZEB family of transcription factors. This molecule plays an important role in regulating cell differentiation and tissue specificity 15. Studies suggest that ZEB1 is expressed in cells from different sources, including bone cells, smooth muscle cells, nerve cells, and T cells 16. In recent years, more and more studies have shown that ZEB1 as a regulator of EMT plays a vital role in the development of tumors. In the process of regulating tumor EMT, ZEB1 can inhibit the expression of epithelial-like gene E-cadherin and miR-200 family members 17. Moreover, ZEB1 is abnormally expressed in different types of tumors, including cervical cancer, pancreatic cancer, osteosarcoma, lung cancer, liver cancer, gastric cancer, colorectal cancer, and breast cancer 18. It is worth noting that the role of ZEB1 in tumor treatment resistance has also begun to be recognized. Studies have found that the expression of ZEB1 has a certain relationship with chemotherapy-resistant tumor cell subtypes 19. Furthermore, silencing the expression of ZEB1 in malignant gliomas not only reduces the invasion ability of tumor cells but also reduces the resistance of tumor cells to the chemotherapy drug temozolomide 19. In pancreatic cancer cells, ZEB1 expression is closely related to the resistance of tumor cells to the chemotherapy drugs gemcitabine, 5-FU uracil, and cisplatin 20. Although people have realized that ZEB1 may play an important role in tumor resistance, the specific mechanism of ZEB1 in colorectal cancer is still unclear and needs further research to clarify.

In this study, we had investigated the expression of USP43 in online databases, clinical samples, and cell lines. The functional roles of USP43 in cell lines had been further studied. Moreover, the detailed molecular mechanisms of USP43 and ZEB1 were reported. The information provided in this study could provide valuable clues to further study in lung adenocarcinoma.

## Materials and method

### Tissue samples and cells

50 colorectal cancer samples and their paired normal tissues were collected in the Department of pathology, Fujian Medical University Union Hospital between Jan 2019 and Dec 2019. The inclusion criteria were right hemi-colon, left-hemicolon and rectum carcinoma specimen. The exclusion criteria were: 1. Active secondary malignancies; 2. Start of anti-tumor treatment within 4 weeks before study entry. The ethics committee of Fujian Medical University Union Hospital had reviewed and approved all experimental protocols. All patients had read and signed the informed consent. The detached tissues were quickly frozen with fluid nitrogen and stored at -80℃. FHC, HCT116, SW480, DLD1, and LOVO cells were purchased from ATCC (Virginia, USA). Cells were cultured with RPMI 1640 with 10% (v/v) FBS (Invitrogen, Carlsbad, CA) in a humidified chamber at 5% CO2, at 37°C. SW480 cells were plated on six-well plates (5×10^5^ cells per well). DMEM with 10% FBS without penicillin and streptomycin overnight was used as a culture medium. OPTI-MEM serum-free medium (M5650, Sigma Aldrich) and Lipofectamine 2000 reagent (Thermo Fisher Scientific, USA) was used in transfection tests. The final concentration of 100 nM siRNA was introduced in this study. Meanwhile, pEZ-Lv201 Vector (Biovector, China) was employed to construct the USP43 over-expression system in DLD1 cells. pEZ-Lv201 Vector was used as the negative control in normal DLD1 cells. Lentiviral particles generated with a standardized protocol were used to produce the highly purified plasmids. Endo Fectin-Lenti^TM^ and Titer Boost^TM^ reagents (CWBio, China) were used to co-transfect DLD1 cells. The supernatant was collected after 48 h transfection and stored at -80°C.

### qRT-PCR analysis

Total RNA was extracted with M5 SuperPure Total RNA Extraction Reagent (SuperTRIgent) (mei5bio, China). The mRNA expression was examined with Q225 system (kubotechnology, China). The PCR reaction contained: 10 μL GoldStar Probe Mixture (Low ROX) (CWBio, China), 1 μL sense primer (10 nM), 1 μL anti-sense primer (10 nM), 2 μL cDNA template (10 ng), and 6 μL H_2_O. The program qRT-PCR was set as follows: 95°C, 30 seconds, 40 cycles (95°C, 5 seconds, and 60°C, 10 seconds). 2-ΔΔCt cycle method was used to calculate the relative expression level of mRNAs. GAPDH was employed as the internal control. Primer sequences used in this study were listed in Supplementary Table 1.

### Western blot analysis

Cellular protein in different groups was extracted with 1% PMSF a RIPA Lysis and Extraction Buffer (Beyotime, China). After the total protein was reacted with SDS-PAGE test buffer, sodium dodecy lsulfate-polyacrylamide gel electrophoresis was used to perform further examination. In this step, the proteins were transmembrane onto a polyvinylidene difluoride layer (Novus, USA). After being blocked for 1h at room temperature, the layer was brooded with anti-Rabbit USP43 (1:1000) (NBP2-88562, Novus Biologcials, USA), E-cadherin (1:1000) (#3195S, CST, USA), Vimentin (1:1000) (#5741S, CST, USA), N-cadherin (1:1000) (#13116S, CST, USA), CD133 (1:1000) (#64326, CST, USA), CD44 (1:1000) (#37259S, CST, USA), ZEB1 (1:1000) (#70512S, CST, USA), GAPDH (1:1000) (#2118, CST, USA), overnight. Proteins were hatched with the corresponding secondary antibodies for 1 h at room temperature after treated with ECL Chemiluminescence Detection Kit (PromoCell, German). The bands were observed with Chemiluminescence Imaging (clinx Ltd., China).

### Immunohistochemical staining analysis

The immunohistochemical SP method was used to stain cancer tissue sections. Tissue sections were baked in a 60°C incubator for 1 h. The tissue sections were subjected to multiple treatments, including immersion in xylene to dewax, gradient alcohol hydration, microwave antigen repair, and 3% hydrogen peroxide treatment. After the goat serum was blocked, an anti-rabbit TRIM2 monoclonal antibody USP43 (1:1000) (NBP2-88562, Novus Biologcials, USA) was added and incubated at 4°C overnight. The section was observed under an optical microscope.

### Migration and Invasion assay

EZCell™ Cell Migration/Chemotaxis Assay Kit (24-well) (K911-12, Biovision, USA) and EZCell™ Cell Invasion Assay (Basement Membrane) (96-well Kit) (K912-100, Biovision, USA) were used to performing cell migration assay and cell invasion, respectively. The detailed steps were strictly followed by the instruction provided by the manufacturer.

### CCK8

The differently treated cells were digested, centrifuged, and resuspended. The cells were diluted with complete medium. We counted using a cell glass counting plate, and then diluted the cells to 2000 cells/ml. 100 μL of a 2000 cells/ml cell suspension was added to each well in a 96-well plate. There are 5 replicate wells in each group. Five replicates were set and observed in five-time points. Subsequently, we incubated the cells in a 5% CO2, 37°C incubator overnight. The next day, 10 μL of CCK-8 solution (Beyotime, China) was added to the medium of the first 5 pairs of wells. Then, the plate was incubated in a 37°C incubator for 2 h. The absorbance at OD450 was measured after the crystals were thoroughly dissolved. Then, cell proliferation was calculated.

### Cell pelleting

We used DMEM/F12 medium containing 20 ng/mL of EGF, 20 ng/mLbFGF and B27 for cell pelleting experiments. We resuspended and counted the different TRIM2 treated cells. 1 mL of medium and 200 cells were added to a low-adhesion 24-well plate. 100 μL of the above-mentioned medium was added every one day. After two weeks, the picture was taken under a microscope. The diameter of the cell pellet was measured and counted.

### Immunofluorescence analysis

Cancer cells in the logarithmic growth phase were inoculated into 24-well plates with cell slides and cultured for 48 h. We discarded the medium, removed the cell slides, and washed 3 times with PBS. Sections were fixed with 4% paraformaldehyde at 4°C for 30 minutes. We have washed 3 times at room temperature with PBS for 5 minutes each. Furthermore, 0.1% Triton was used to treat sections for 10 min and PBS was used to wash sections for 5 min. The goat serum incubation section was blocked for 1 h at room temperature. The goat serum incubation section was blocked for 1 h at room temperature. Subsequently, we added a diluted secondary antibody, reacted for 1 h at room temperature in a wet box, and washed 3 times with PBS for 10 min each. We use an inverted fluorescence microscope to observe the results.

### Co-IP detection

In this study, we use cancer cells in logarithmic growth phase. Total protein was extracted using the RIPA Lysis and Extraction Buffer (89900, ThermolFisher Scientific, USA). In short, we washed the beads with 100 μL of ice-cold buffer. We added 100 μL of antibody binding buffer to spin the antibody and magnetic beads for 30 min. We washed the beads 3 times with 200 μL of buffer for 5 min each. We used cell lysate and antibody-conjugated magnetic beads to incubate for 1 h at room temperature and washed the beads 3 times with 200 μL buffer for 5 min each. 20 μL of elution buffer was used to wash the beads once and the supernatant was taken.

### Scratch test

We resuspended and counted different ZEB1 knockdown treated cells. The scratch test insert after alcohol disinfection was carefully placed in a 12-well plate (3 replicates per group). We used the complete medium to dilute the cells to 500 cells/μL. 70 μL of the cell suspension was added to each well. Place at 37°C (incubate in a 5% CO2 incubator for 24 h). After 24 h, we gently washed the cells twice with PBS. Then, 1 ml of 1% FBS corresponding medium was added. Cell status was observed under the microscope at 0h and 24 h.

### Statistical methods

In this study, SPSS16.0 statistical software was used. The data were expressed as χ ± s. Two groups were compared using the *t*-test. One-way analysis of variance was used for comparison between groups. *P* values < 0.05 were considered to be significant differences between the two data.

## Results

### USP43 gene is highly expressed in lung adenocarcinoma tissue

**Figure 1:**
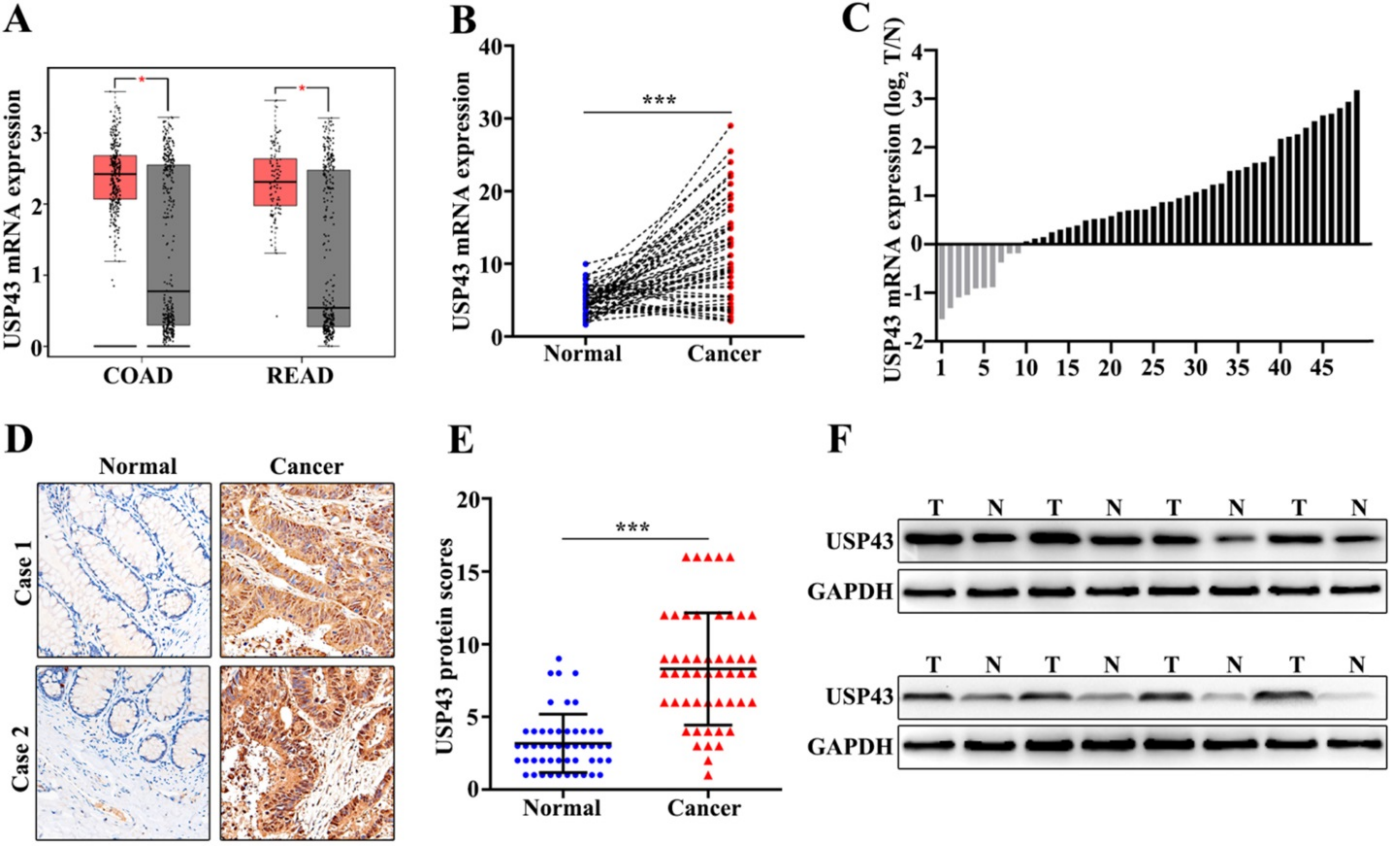
** Detection of USP43 expression in colorectal cancer. A.** The expression level of USP43 was verified in GEPIA database (http://gepia.cancer-pku.cn/). **B.** qRT-PCR analysis of USP43 expression level in colorectal cancer tissues and normal tissues. **C.** The sample distribution analysis of the high expression in tumor tissue and high expression in adjacent tissues among 50 pairs of specimens. **D.** Detection of USP43 expression levels in colorectal cancer tissues and normal tissues with IHC. **E.** IHC score statistics of the USP43 expression levels in 50 colorectal cancer tissues and normal tissues. **F.** Western blot analysis of the USP43 expression level in colorectal cancer tissues and normal tissues. *P<0.05, ***P<0.001.

In this study, we have included 50 CRC patients. The clinical features of the 50 patients were shown in Table 1. The results suggested that significant differences could be calculated in T Stages (*P*=0.001) and Metastasis (*P*=0.001). Moreover, we have examed the expression of USP43. Firstly, we had detected the expression of USP43 in colorectal cancer tissue and the paired normal tissues with the online dataset, western blot, qRT-PCR, and immunohistochemical staining analysis. For online dataset analysis, we evaluated USP43 expression in GEPIA database (http://gepia.cancer-pku.cn/index.html). USP43 expression in colorectal cancer tissues was higher than those in the paired normal tissues (*P*<0.05) (Figure 1A). Meanwhile, qRT-PCR analysis indicated that USP43 expression in colorectal cancer tissues was significantly higher than that in the paired normal tissues (*P*<0.001) (Figure 1B). Moreover, we have analyzed the distribution of the high USP43 expression in colorectal cancer tissue and the paired adjacent tissues. Figure 1C suggested that 80% (40 of 50) high USP43 expression could be detected in colorectal cancer tissues. Furthermore, immunohistochemical staining analysis indicated that USP43 expression in colorectal cancer tissue was significantly higher than that in the paired normal tissues (*P*<0.001) (Figure 1D and Figure 1E). In addition, western blot analysis revealed that USP43 protein expression in colorectal cancer tissues was significantly higher than normal tissues. In summary, USP43 expression in colorectal cancer tissue was higher than those in the paired normal tissues.

### USP43 promotes proliferation of colorectal cancer cells *in vitro*

**Figure 2:**
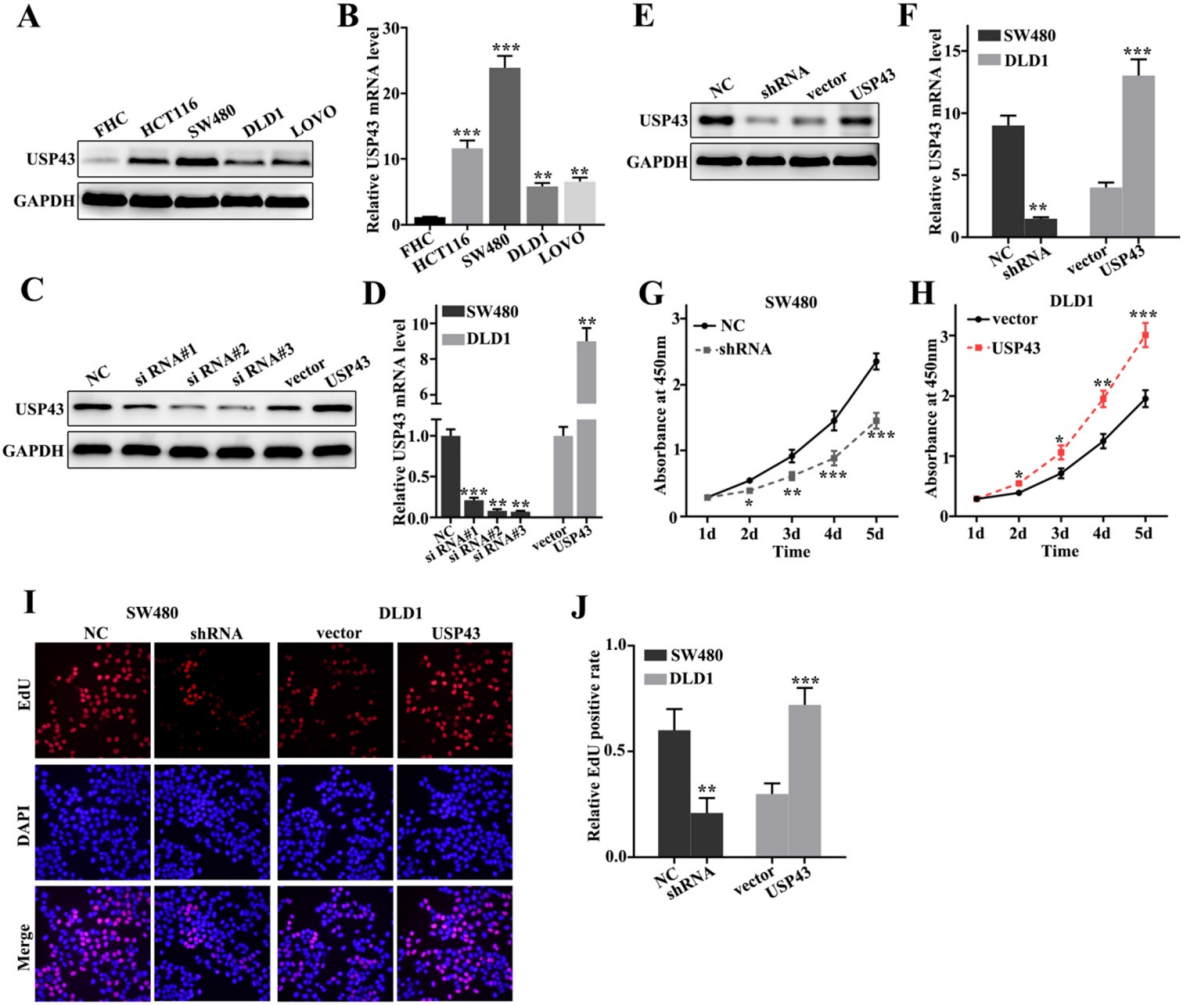
** USP43 promotes colorectal cancer cell proliferation *in vitro.* A-B.** Western blot and qRT-PCR analysis of the USP43 protein and mRNA levels in normal colon epithelial cell lines and four common colorectal cancer cells. **C-D.** Western blot and qRT-PCR were used to select sequences with better knockdown efficiency. **E-F.** Western blot and qRT-PCR were used to identify the effects of USP43 knockdown treatment in SW480 cells and USP43 overexpression treatment in DLD1 cells. **G-J.** CCK8 and edu experiment was employed to study the cell proliferation of USP43 knockdown treated SW480 cells and USP43 overexpression treated DLD1 cells. *P<0.05, **P<0.01, ***P<0.001.

To further probe the biological function of USP43 in colorectal cancer cells, we have studied USP43 expressions in five selected cell lines, including FHC, HCT116, SW480, DLD1, and LOVO. Western blot analysis and qRT-PCR analysis of USP43 expression in five cell lines indicated that USP43 protein and mRNA expressions were significantly different between each other (Figure 2A and Figure 2B). It was notable that USP43 protein and mRNA expression in DLD1 cells were lower than those in other cell lines (*P*<0.01). Meanwhile, USP43 protein and mRNA expression in SW480 cells were higher than those in other cell lines (*P*<0.001). Therefore, DLD1 and SW480 cells were selected to carry out further study. For DLD1 and SW480 cells, we performed overexpression and knockdown of USP43 treatments, respectively. We have employed western blot and qRT-PCR analysis to examine the USP43 protein and mRNA expression in DLD1 and SW480 cells with different treatments. Figure 2C and Figure 2D showed that overexpression and knockdown of USP43 treatments in DLD1 and SW480 cells were successful. Si RNA #3 and USP43 vector were employed to further study. Meanwhile, we have identified the efficiency of overexpression and knockdown treatment in USP43 knockdown treated SW480 cells, and USP43 overexpression treated DLD1 cells. Figure 2E and Figure 2F revealed that overexpression and knockdown system used in this study were two effective systems. For cell proliferation analysis, USP43 knockdown in SW480 cells could significantly reduce cell proliferation compared with those in normal SW480 cells on days 2, 3, 4, 5 (Figure 2G) (*P*<0.01). However, USP43 overexpression in DLD1 cells could significantly promote cell proliferation compared with those in vector treated DLD1 cells on days 2, 3, 4, 5 (Figure 2F) (*P*<0.05). Moreover, we have further employed edu experiment to probe the cell number changes in USP43 knockdown in SW480 cells and USP43 overexpression in DLD1 cells. Figure 2I and Figure 2J suggested that similar results of CCK-8 could be observed in edu experiment. USP43 knockdown in SW480 cells could significantly reduce cell proliferation. Meanwhile, USP43 overexpression in DLD1 cells could significantly promote cell proliferation *in vitro*.

### Regulation of USP43 on lung adenocarcinoma cell migration and invasion

**Figure 3:**
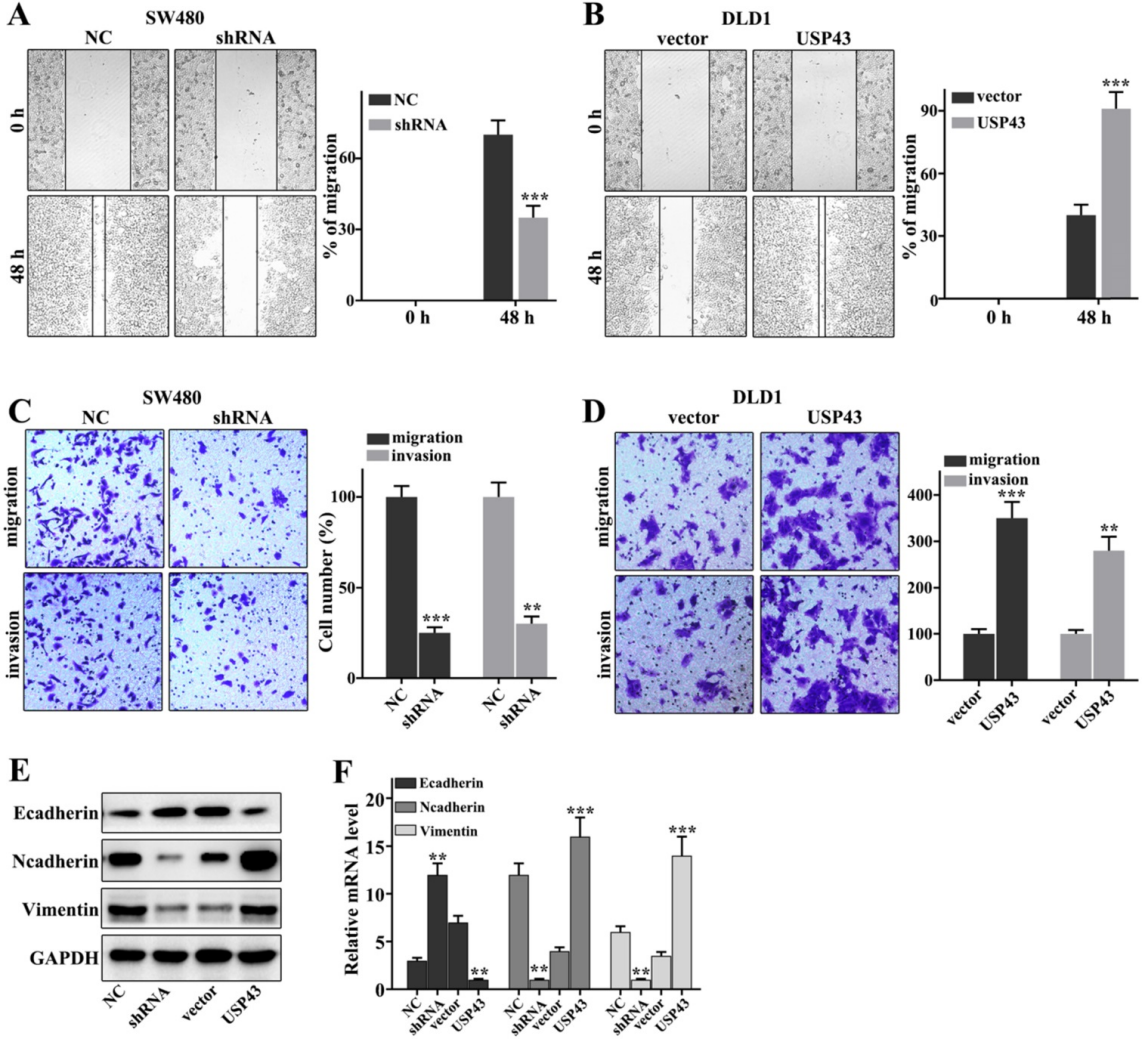
** USP43 regulates the migration and invasion of colorectal cancer cells. A-D.** Cell scratch test, migration, and invasion assays analysis of USP43 knockdown treated SW480 cells and USP43 overexpression treated DLD1 cells. **E-F.** Western blot and qRT-PCR analysis of the EMT-related proteins (Ecad Ncad, and Vim in USP43 knockdown treated SW480 cells and USP43 overexpression treated DLD1 cells. **P<0.01, ***P<0.001.

In this work, we have investigated the regulation of USP43 on colorectal cancer cells migration and invasion in USP43 knockdown in SW480 cells and USP43 overexpression in DLD1 cells. Figure 3A showed that USP43 knockdown in SW480 cells could significantly inhibit cell scratch ability compared with those in normal SW480 cells (*P*<0.001). Meanwhile, USP43 overexpression in DLD1 cells could significantly promote cell scratch ability compared with those in normal DLD1 cells (*P*<0.01) (Figure 3B). Moreover, we have further examined the cell migration and invasion in USP43 knockdown in SW480 cells and USP43 overexpression in DLD1 cells. USP43 knockdown in SW480 cells could significantly inhibit cell migration and invasion compared to that in the normal SW480 cells (*P*<0.01) (Figure 3C). However, USP43 overexpression in DLD1 cells could promote cell migration and invasion compared to that in the normal DLD1 cells (*P*<0.01) (Figure 3D). In addition, we have further analysis of the protein expressions of E-cadherin, N-cadherin, and Vimentin. USP43 knockdown in SW480 cells could effectively inhibit protein expressions of N-cadherin and Vimentin (*P*<0.01) (Figure 3E and Figure 3F). However, USP43 knockdown in SW480 cells could effectively promote protein expressions of E-cadherin (*P*<0.01). Moreover, USP43 overexpression in DLD1 cells could effectively increase protein expressions of N-cadherin and Vimentin (*P*<0.01) (Figure 3E and Figure 3F). However, USP43 overexpression in DLD1 cells could effectively inhibit E-cadherin protein expressions (*P*<0.01). Therefore, USP43 overexpression in DLD1 cells could promote and inhibit the protein expressions of N-cadherin and Vimentin and protein expressions of N-cadherin, respectively. Meanwhile, USP43 knockdown in SW480 cells could inhibit and promote the protein expressions of N-cadherin and Vimentin and protein expressions of N-cadherin, respectively.

### Effect of USP43 on chemotherapy sensitivity of lung adenocarcinoma cells

**Figure 4:**
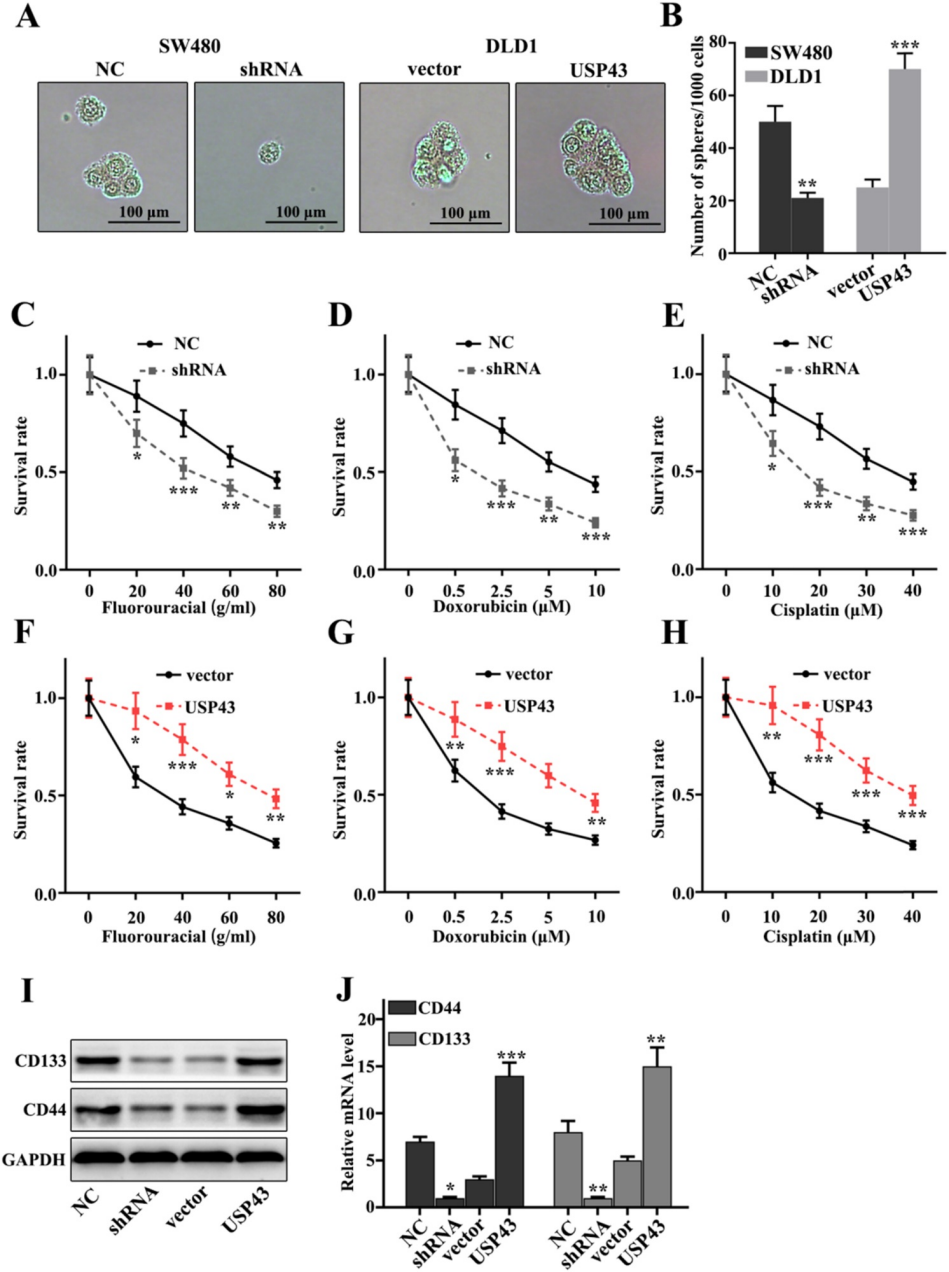
** Effect of USP43 on chemotherapy sensitivity of colorectal cancer cells. A-C.** Stem cell ball culture analysis of USP43 knockdown treated SW480 cells and USP43 overexpression treated DLD1 cells. **D.** Cellular chemotherapy sensitivity experiment on three common chemotherapeutics. **I-J.** Western blot and qRT-PCR analysis of stem cell-related proteins (CD133/CD44). *P<0.05, **P<0.01, ***P<0.001.

In this study, we have investigated the effect of USP43 expression changes on chemotherapy sensitivity of colorectal cancer cells. Cell pelleting analysis showed that USP43 knockdown treatment in SW480 cells and USP43 overexpression treatment in DLD1 cells could significantly inhibit and promote cell pelleting ability compared with that in control groups (*P*<0.01) (Figure 4A and 4B). Meanwhile, we have employed three common chemotherapeutic molecular drugs, including fluorouracial, doxorubicin, and cisplatin to probe the survival rate of USP43 knockdown in SW480 cells and USP43 overexpression in DLD1 cells. Figure 4C, Figure 4D, and Figure 4E showed the survival rate of USP43 knockdown treated SW480 cells. The results suggested that USP43 knockdown treated SW480 cells could significantly decrease cell survival in three different drug treatments compared with that in normal SW480 cells (*P*<0.05). However, USP43 overexpression in DLD1 cells could effectively promote cell survival in three different drug treatments compared with that in normal DLD1 cells (*P*<0.05) (Figure 4F-H). These results revealed that USP43 knockdown treatment in SW480 cells and USP43 overexpression treatment in DLD1 cells was closely related to the changes in cell survival rate caused by drug treatments. Moreover, we have evaluated the protein and mRNA expression of stem cell-related biomarkers, including CD44, and CD133. Figure 4I and Figure 4J indicated that USP43 knockdown treatment in SW480 cells could significantly decrease protein and mRNA expression of stem cell-related biomarkers (*P*<0.001). However, USP43 overexpression in DLD1 cells could effectively promote protein and mRNA expression of stem cell-related biomarkers (*P*<0.001). The above results suggested that USP43 expression was closely related to stem cell ability.

### USP43 regulates ZEB1 expression through ubiquitination

**Figure 5:**
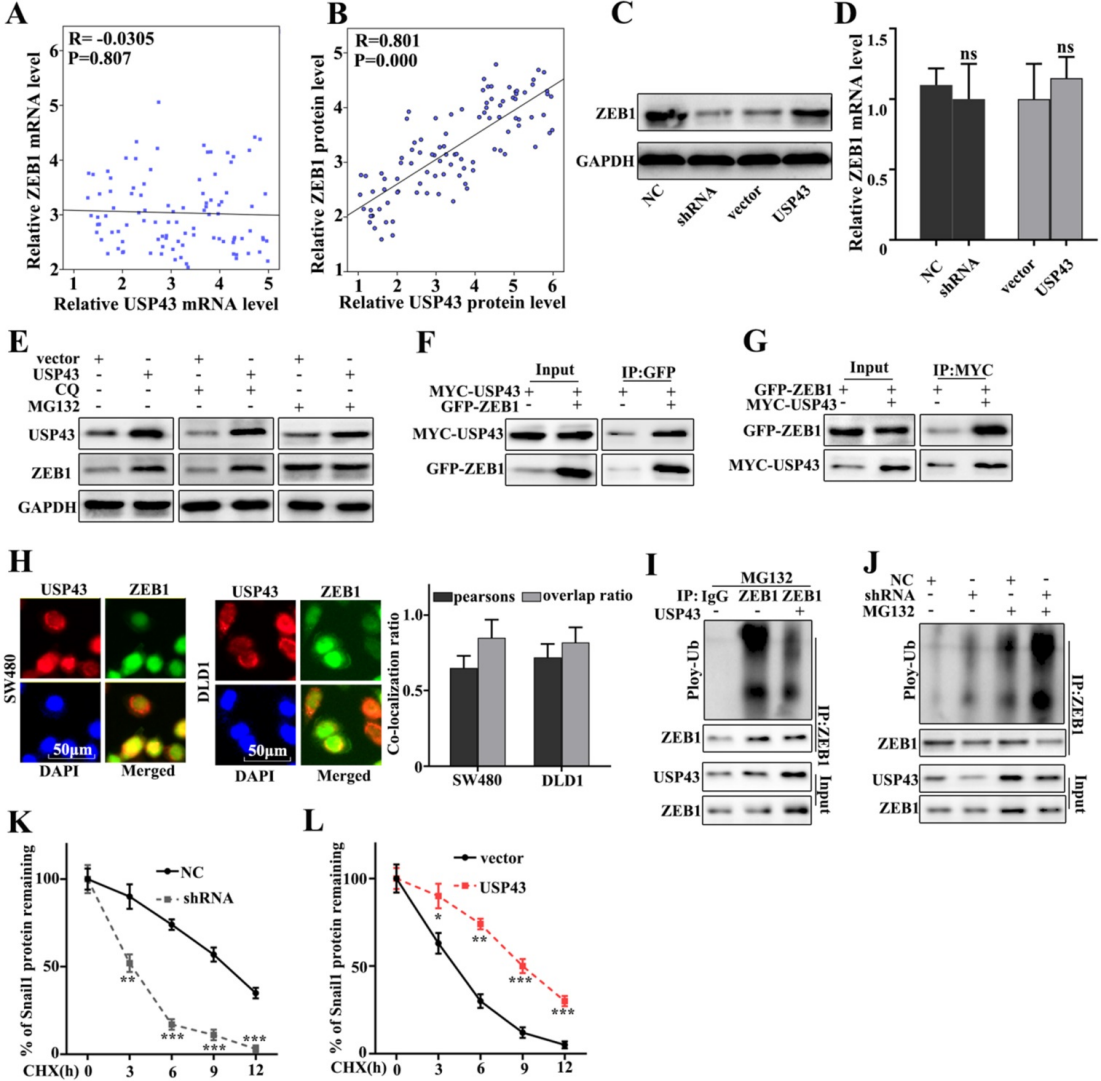
** USP43 regulates ZEB1 expression through ubiquitination. A.** Verifying the correlation between USP43 and ZEB1 mRNA in 50 colon cancer specimens. **B.** The correlation between USP43 and ZEB1 protein expression was calculated In 50 pairs of colon cancer specimens. **C-D.** Western blot and qRT-PCR analysis the relationships between USP43 and ZEB1. **E.** ZEB1 protein expression analysis with CQ and MG132 treatments. **F.** Co-precipitation analysis of USP43 and ZEB1 protein in SW480 cell **G.** co-localization of immunofluorescence analysis of USP43 and ZEB1 in SW480 and DLD1 cell. **H.** Co-precipitation of USP43 and ZEB1 in the 293T cell. **I.** Ubiquitination testing of USP43 in DLD1 cell line. **J.** Ubiquitination testing of USP43 in SW480 cell line. **K-L.** Cycloheximide (protein degradation rate) analysis shows that USP43 can inhibit ZEB1 protein degradation. *P<0.05, **P<0.01, ***P<0.001.

In this study, we have further studied the potential relationships between the expressions of USP43 and ZEB1. We have further analyzed the potential relationships of the mRNA expressions of USP43 and ZEB1 in 50 clinical samples (Figure 5A and Figure 5B). The results suggested that no correlation could be calculated between the mRNA expressions of USP43 and ZEB1 in clinical samples. However, significant correlation could be calculated between the protein expressions of USP43 and ZEB1 in clinical samples (*P*=0.000). Moreover, western blot and qRT-PCR were employed to investigate the protein and mRNA expressions of ZEB1 in USP43 knockdown treated SW480 cells, and USP43 overexpression treated DLD1 cells. Figure 5C showed that ZEB1 protein expression in USP43 knockdown treated SW480 cells was lower than that in normal SW480 cells. However, ZEB1 protein expression in USP43 overexpression treated DLD1 cells were more abundant compared with that in normal DLD1 (Figure 5C). It was notable that no difference of USP43 mRNA abundance could be detected in USP43 knockdown treated SW480 cells and USP43 overexpression treated DLD1 cells (Figure 5D). The above results suggested that USP43 could affect the ZEB1 protein expression but not Snail1 mRNA abundance. Moreover, we have investigated the potential relationships between USP43 ubiquitination and ZEB1 in USP43 overexpression DLD1 cells. MG132 is the inhibitor of the proteasome degradation pathway in the cell. Meanwhile, Chloroquine (CQ) is an inhibitor of the autophagolysosomal degradation pathway. Therefore, we have employed MG132 (25 Um) and CQ (25 Um) to study the protein expressions of USP43 and ZEB1 in USP43 overexpression DLD1 cells (Figure 5E). The results suggested that MG132 can effectively increase ZEB1 protein expression. However, CQ treatment did not affect ZEB1 protein expression. Therefore, we speculate that USP43 could regulate ZEB1 expression through the ubiquitination but not the cellular autophagy pathway. Moreover, we have examined the potential interaction between USP43 protein and ZEB1 protein in cellular. In this study, Co-immunoprecipitation (Co-IP) was employed to study the potential interaction between USP43 protein and ZEB1 protein. Figure 5F showed that USP43 protein could interact with ZEB1 protein. Moreover, we have carried out the forward and reverse protein Co-IP of USP43 protein and ZEB1 protein in USP43 overexpression DLD1 cells. Figure 5F and 5G had further identified the interaction between USP43 protein and ZEB1 protein. Furthermore, immunofluorescence colocalization analysis of USP43 protein and ZEB1 protein revealed that the spatial distribution of USP43 protein and ZEB1 protein is overlapping. The above result further illustrates the mutual combination between USP43 protein and ZEB1 protein in USP43 overexpression DLD1 cells (Figure 5H). In addition, we have further employed ubiquitin detection in USP43 knockdown treated SW480 cells, and USP43 overexpression treated DLD1 cells (Figure 5I and 5J). The results suggested that USP43 knockdown treated SW480 cells could significantly promote the degradation of the remaining ZEB1 protein in cells compared to that normal SW480 cells (*P*<0.01). Meanwhile, USP43 overexpression treated DLD1 cells could significantly decrease the degradation of the remaining ZEB1 protein in cells compared to that normal DLD1 cells (*P*<0.01). Furthermore, we have further employed cycloheximide (CHX), which could to inhibit protein synthesis to study the relationships between protein expressions of USP43 and ZEB1 in USP43 knockdown treated SW480 cells and USP43 overexpression treated DLD1 cells. Figure 5K and 5L suggested that USP43 overexpression in DLD1 cells could significantly promote the remaining ZEB1 protein in cells compared to that normal DLD1 cells (*P*<0.01). Meanwhile, USP43 knockdown treated SW480 cells could significantly decrease the remaining ZEB1 protein in cells compared to that normal SW480 cells (*P*<0.01).

### Rescue experiment

**Figure 6:**
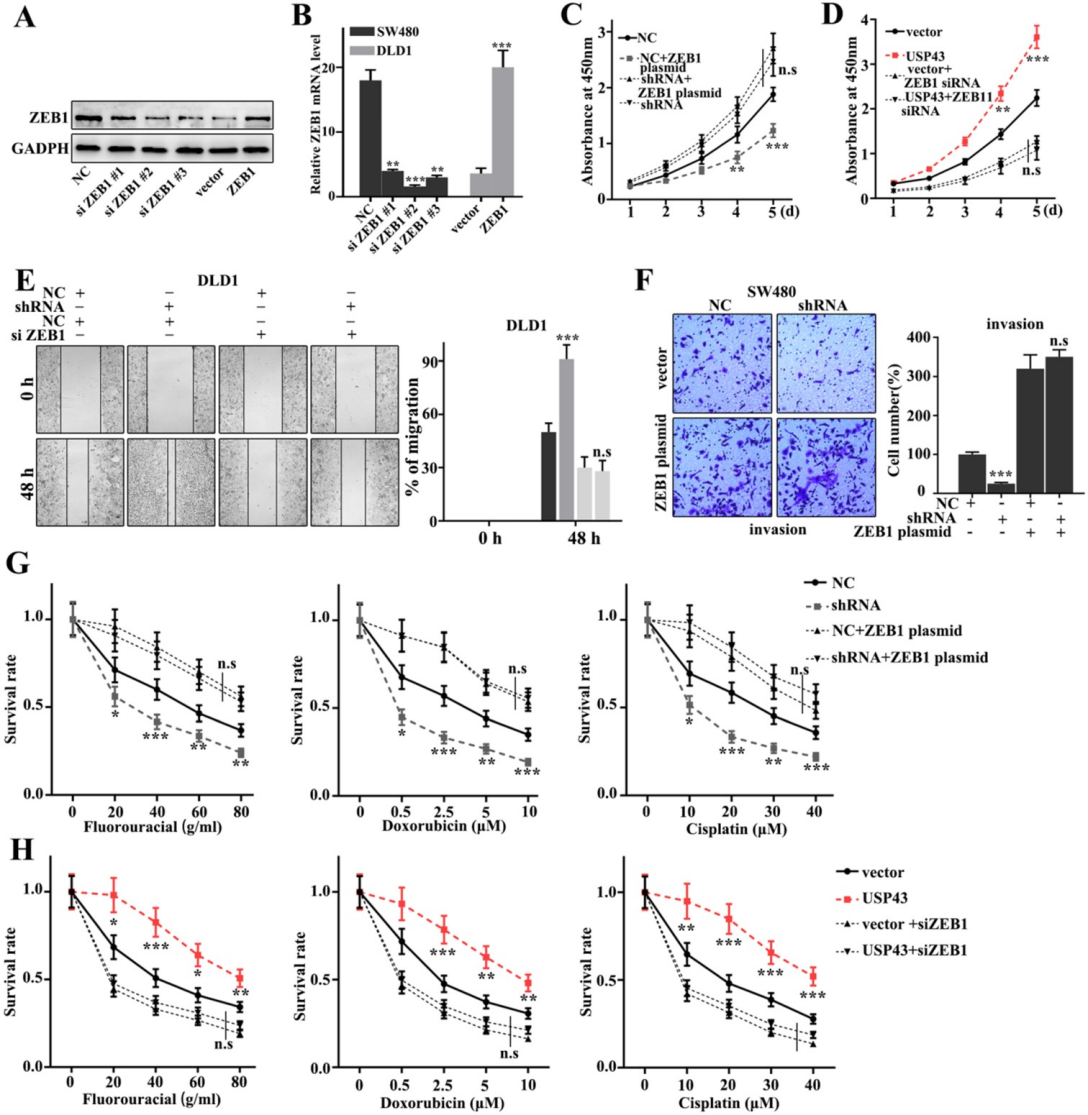
** Rescue experiment. A-B.** Western blot and qRT-PCR analysis of the optimal interference sequence and overexpression sequence of ZEB1 siRNA. **C-D.** CCK8 analysis of the USP43 knockdown treated SW480 cells and USP43 overexpression treated DLD1 cells with ZEB1 siRNA and overexpression plasmids treatments. **E.** Scratch experiment analysis of the USP43 overexpression treated DLD1 cells with ZEB1 siRNA and overexpression plasmid treatments. **F.** Migration ability analysis of USP43 knockdown treated SW480 cells with ZEB1 siRNA and overexpression plasmid treatments. **G-H.** Three common chemotherapeutics for cell survival rate analysis of the USP43 knockdown treated SW480 cells and USP43 overexpression treated DLD1 cells with ZEB1 siRNA and overexpression plasmids treatments. *P<0.05, **P<0.01, ***P<0.001.

In this study, we have further performed the rescue experiment to demonstrate the relationships between USP43 and ZEB1. We have further introduced ZEB1 knockdown and over-expression treatment in USP43 knockdown treated SW480 cells and USP43 overexpression treated DLD1 cells. Figure 6A and 6B showed that siZEB1 #3 and ZEB1 vector were effective experimental systems to knockdown and over-expression treatment of ZEB1 gene protein and mRNA in normal SW480 cells and DLD1 cells, respectively. Meanwhile, Figure 6C and 6D suggested that knockdown and over-expression treatment of ZEB1 gene treatments could effectively inhibit and promote cell proliferation caused by USP43 overexpression and knockdown treatments, respectively. Moreover, we have studied the cell scratch of the ZEB1 knockdown and over-expression treatments in DLD1 cells, which had been treated with USP43 overexpression and USP43 knockdown, respectively. Figure 6E showed that the ZEB1 knockdown could effectively reverse the changes in cell scratch caused by USP43 knockdown (*P*<0.01). Meanwhile, ZEB1overexpression could effectively reverse the changes in invasion caused by USP43 overexpression in SW480 cells (*P*<0.01) (Figure 6F). In addition, we have studied the effect of USP43 and ZEB1 on chemotherapy sensitivity of colorectal cancer. Figure 6G and Figure 6H suggested that ZEB1 over-expression and knockdown treatments could effectively reverse the changes in cell survival rate caused by USP43 knockdown and overexpression treatment in the chemotherapy sensitivity analysis (*P*<0.01). In summary, knockdown and overexpression of ZEB1 could effectively rescue the changes in cell proliferation, migration, invasion, and EMT in USP43 overexpression and knockdown treated cells.

## Discussion

Colorectal cancer is a common malignant tumor that seriously threatens human life and health. The etiology of this disease has not yet been clarified. Genetics, diet, unhealthy lifestyles, and precancerous lesions are reported to be closely related to its occurrence 21. Meanwhile, more than half of colorectal cancer patients in clinical practice have undergone radical surgery before micrometastasis, which is the direct cause of postoperative metastasis and recurrence of colorectal cancer 22. The difficulty in diagnosis and treatment of colorectal cancer is one of the important reasons for its high morbidity and mortality 23. Therefore, the research on the mechanism of the development of colorectal cancer is particularly important. It can not only help to understand the pathogenesis of lung adenocarcinoma, but also assist in the development of anti-tumor drugs.

Ubiquitination is one of the important post-translational modifications of proteins, which plays an important role in regulating cell proliferation, differentiation, and apoptosis 24. Previous articles have demonstrated the role of ubiquitin ligase FBXW7 in regulating tumor metastasis and epithelial-mesenchymal transition 25. Similarly, the process of deubiquitination by hydrolyzing ubiquitin and stabilizing whip protein has also received increasing attention 26. Ubiquitin-specific proteases (USPs) are the largest family of deubiquitinase. It has been reported that a variety of USP plays a role in regulating cancer development, such as USP7 27 and USP33 28. In this study, our results suggested that the change of USP43 expression was closely related to the invasion, migration, proliferation, tumor stemness, and tumor stem cell spheroidizing ability of CRC cells. Therefore, USP43 may provide a potential target for the treatment of CRC. Recent reports indicated that the abnormal expression of USP43 in CRC and breast cancer tissues is associated with a poorer prognosis 14, 29. Those results are consistent with our study. Although the underlying mechanism of USP43 in CRC patients may be complex and diverse, it is still an important gene involved in tumorigenesis and development. This study showed that the expression of USP43 in CRC tissues was higher than in adjacent tissues. Overexpression of USP43 or knockout of USP43 can affect the proliferation, migration, and stemness of lung adenocarcinoma cells. Therefore, it is suggested that USP43 dysfunction may be one of the factors affecting the progression of CRC.

The transcription factor ZEB1 is encoded by the TCF8 gene and can bind to the E-box sequence CAGGTG/A and regulate gene expression 30. Previous studies have focused on ZEB1 inhibition of target genes. However, there is increasing evidence that ZEB1 can activate target gene transcription 31. In tumor research, ZEB1 is closely related to the occurrence and development of various tumors. In prostate cancer, ZEB1 overexpression has become an important marker molecule for metastasis assessment 32. In renal clear cell carcinoma, ZEB1 promotes tumorigenesis 33. In endometrial cancer, ZEB1 may cause the down-regulation of E-cadherin expression and eventually lead to enhanced tumor cell invasion and migration ability 34. In breast cancer, ZEB1 promotes the malignant progression of tumors and makes the cells exhibit stromal-like characteristics 35. Meanwhile, ZEB1 has been considered to be an important EMT-related transcription factor, which plays an important role in the induction and maintenance of a variety of interstitial-like features 36. Recent studies have reported that ZEB1 can also induce breast cancer resistance to radiotherapy 37, which is also an important reason for the resistance of tumor cells to chemotherapy 38. In this study, it was notable that ZEB1 (EMT biomarker) expression was significantly affected in USP43 overexpression and knockdown treated cells. ZEB1 can directly interact with USP43 in cellular. Moreover, USP43 can reduce ZEB1 protein expression without affecting its transcription. Moreover, our results suggested that USP43 does affect the protein degradation pathway of ZEB1 through ubiquitination modification. Meanwhile, USP43 overexpression and knockdown treatments with knockdown and overexpression of ZEB1 could affect cell migration, invasion, and EMT-related biomarkers such as E-caderin, N-caderin, and Vitmentin. Therefore, our results revealed that USP43 and ZEB1 can regulate EMT of colorectal cancer.

## Conclusion

In summary, we demonstrated that high USP43 expression could be detected in colorectal cancer tissues and cells. USP43 could promote cell proliferation, invasion, and migration in colorectal cancer by regulating ZEB1 ubiquitylation degradation. Our results could provide detailed information for further studies in colorectal cancer.

## Supplementary Material

Supplementary table S1.

## Figures and Tables

**Table 1:** Clinical features of the patients included in this study

Features	Total (n)	USP43			
		Positive	Negative	X^2^	*P*-value
**Gender**					
Male	30	26	4	2.083	0.149
Female	20	14	6		
**Age (years)**					
≥60	28	24	4	1.299	0.254
<60	22	16	6		
**T Stages**					
I-II	18	10	8	10.503	0.001*
III-IV	32	30	2		
**Metastasis**					
**N Stages**					
N0	10	5	5	7.031	0.008*
N1-2	40	35	5		
**M Stages**					
M0	42	35	7	1.823	0.177
M1	8	5	3		
**Location**					
Colon	28	23	5	0.521	0.47
Rectal	22	17	5		
**Histological Differentiation**					
Well-moderate	29	25	4	1.663	0.197
Poorly	21	15	6		
