# Supplementary material for: USP43 directly regulates ZEB1 protein, mediating proliferation and metastasis of colorectal cancer
Source: J Cancer. 2021 Jan 1;12(2):404–16. doi: 10.7150/jca.48056 (PMC7738986; doi:10.7150/jca.48056)
Supplement: Supplementary file 1 — Supplementary table S1. [file jcav12p0404s1.pdf]

Supplementary table 1. The sequence of primers for Quantitative reverse transcription –PCR.

Gene	Forward primer (5’------3’)	Reverse primer(5’------3’)
USP43	GAGTCAAGTGAAGTGCCAATGT	TGAGGGCAGCTATATGGTCTC
E-cadherin	CGAGAGCTACACGTTCACGG	GGGTGTCGAGGGAAAAATAGG
Vimentin	CGAAACTTCTCAGCATCACG	GCAGAAAGGCACTTGAAAGC
N-cadherin	TCAGGCGTCTGTAGAGGCTT	ATGCACATCCTTCGATAAGACTG
CD133	AGTCGGAAACTGGCAGATAGC	GGTAGTGTTGTACTGGGCCAAT
CD44	CTGCCGCTTTGCAGGTGTA	CATTGTGGGCAAGGTGCTATT
ZEB1	CAGCTTGATACCTGTGAATGGG	TATCTGTGGTCGTGTGGGACT
GAPDH	AGAAGGCTGGGGCTCATTTG	AGGGGCCATCCACAGTCTTC
